# Association between the insulin resistance marker TyG index and subsequent adverse long-term cardiovascular events in young and middle-aged US adults based on obesity status

**DOI:** 10.1186/s12944-023-01834-y

**Published:** 2023-05-18

**Authors:** Weihua Chen, Shan Ding, Jiabin Tu, Guitao Xiao, Kaihong Chen, Yanbin Zhang, Rongchong Huang, Ying Liao

**Affiliations:** 1 Department of Cardiology, Longyan First Affiliated Hospital of Fujian Medical University, Longyan, 364000 China; 2 Zhongshan Hospital (Xiamen), Fudan University, Xiamen, 361015 China; 3 Department of Cardiology, Beijing Friendship Hospital, Capital Medical University, Beijing, 100053 China

**Keywords:** All-cause mortality, Triglyceride-glucose index, Obesity, Cardiovascular events, NHANES

## Abstract

**Background:**

A lthough the triglyceride-glucose (TyG) index has been shown to closely correlate with cardiometabolic outcomes and predict cardiovascular events in many groups, it remains unclear whether obese status in young and middle-aged adults is associated with long-term unfavorable cardiovascular events. This warrants further investigation.

**Methods:**

This retrospective cohort study analyzed data from the National Health and Nutrition Examination Survey spanning the years 1999–2018, with follow-up for mortality status until December 31, 2019. To categorize participants based on the TyG level, the optimal critical value was determined through restricted cubic spline function analysis, dividing them into high and low TyG groups. The study assessed the relationship between TyG and cardiovascular events and all-cause mortality in young and middle-aged adults stratified by obesity status. Kaplan‒Meier and Cox proportional risk models were used to analyze the data.

**Results:**

During a follow-up period of 123 months, a high TyG index increased the risk of cardiovascular events by 63% (*P* = 0.040) and the risk of all-cause mortality by 32% (*P* = 0.010) in individuals after adjusting for all covariates. High TyG was shown to be linked to cardiovascular events in obese people (Model 3: HR = 2.42, 95% CI = 1.13–5.12, *P* = 0.020); however, there was no significant difference in TyG groups for nonobese adults in Model 3 (*P* = 0.08).

**Conclusions:**

TyG was independently associated with harmful long-term cardiovascular events in young and middle-aged US populations, with a stronger association observed in those who were obese.

**Supplementary Information:**

The online version contains supplementary material available at 10.1186/s12944-023-01834-y.

## Introduction

Despite advances in medical technology and research, cardiovascular disease (CVD) is still the major cause of adult deaths in the US [1]. In fact, CVD is responsible for > 1000 fatalities daily [2]. Young adults in the US are experiencing a less dramatic decline in cardiovascular events than older adults [3]. Therefore, a more thorough assessment of the short- and long-term prognoses of CVD in these individuals is needed.

Insulin resistance (IR), a state of peripheral tissue insensitivity to insulin, is known to be a key pathogenic characteristic of type 2 diabetes mellitus (DM) [4, 5]. The triglyceride-glucose (TyG) index, which is the logarithmic product of fasting triglyceride (TG) and glucose concentration, has recently been proposed as a replacement for IR [6, 7]. The TyG index has now been shown to have a strong correlation with cardiometabolic outcomes [8–11] and to be able to predict cardiovascular events in a variety of populations [12].

With an estimated 700 million obese adults worldwide, obesity is a well-known and well-described risk factor for CVD, DM, and other illnesses [13, 14]. There are significant connections between IR, inflammation, and obesity. Moreover, dietary structure and IR or indicators of inflammation are influenced by obesity [15, 16]. Little information is available, however, regarding how the TyG index impacts the prognosis of young and middle-aged community members based on the level of obesity.

The goal of this retrospective study was to ascertain the relationship between cardiovascular incidents and all-cause mortality in young and middle-aged community-dwelling obese and nonobese persons using the National Health and Nutrition Examination Survey (NHANES).

## Methods

### Study design and participants

NHANES was developed and is managed by the National Center for Health Statistics (NCHS) at the Centers for Disease Control and Prevention (CDC). The NHANES study required a full review and ethical approval of the NCHS Research Ethics Assessment Board. The selected participants, or their guardians, were fully informed about the project and provided signed consent after receiving that information. The public can obtain all currently accessible information at the NCHS of the CDC.

**Fig. 1:**
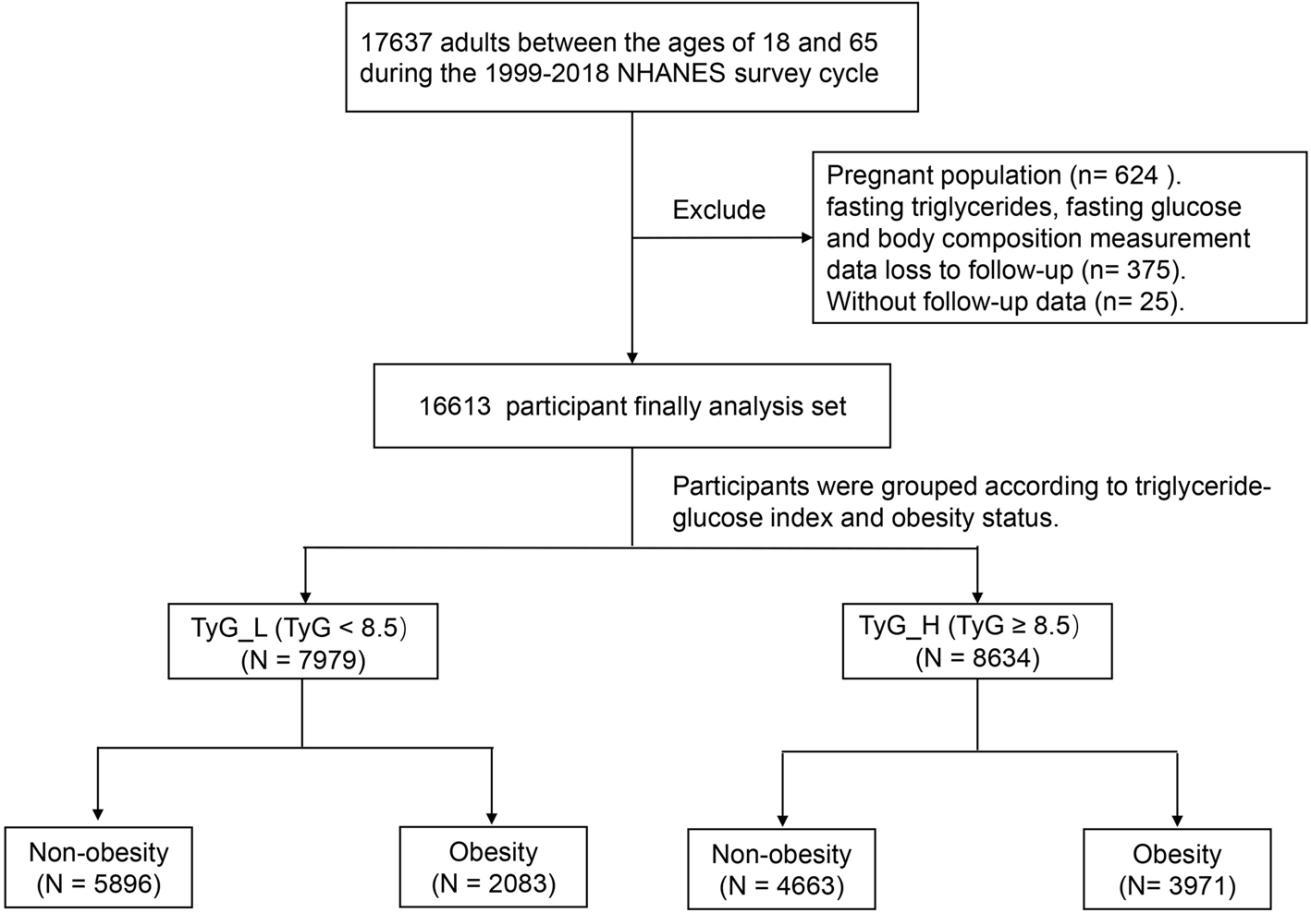
Flowchart

A retrospective study was conducted from the NHANES. According to Fig. 1, the NHANES participants in the U.S. community who were > 18 and 65 years old (*N* = 17,637) were included. Due to the absence of fasting TG, glucose, or body composition measurements, 375 additional prospective participants and 624 pregnant women were eliminated. Twenty-five potential participants were also lost to follow-up. As a result, 16,613 individuals were eventually enlisted for the pertinent analyses in the current study.

### Exposure

The TyG index was derived from the baseline data, and TyG = Ln [fasting TG (mg/dL) × fasting glucose (mg/dL)/2] was used to establish the TyG index [6]. According to typical mobile edge computing practices, weight was determined using a digital scale, and height was determined using a rangefinder. Body mass index (BMI) is a simple and popular indicator of obesity. The optimal critical value of TyG was determined to be 8.5 through restricted cubic spline function analysis. We divided the participants into two TyG categories: < 8.5 for the TyG_L group and ≥ 8.5 for the TyG_H group. The following four groups were then created by combining the TyG index and BMI: 1) TyG_L/nonobesity group; 2) TyG_L/obesity group; 3) TyG_H/nonobesity group; and 4) TyG_H/obesity group, all with BMIs of 30 kg/m^2^.

### Defining variables of interest

The following information was self-reported: age, sex, race, education level, status as a smoker, alcohol use, DM, hypertension, CVD history, and family histories of CVD and DM. Blood was drawn from veins in the patients' arms after they had fasted for more than nine hours to send the samples to the lab for analysis the following morning. Hexokinase was used to measure the fasting plasma glucose (FPG) concentration. The concentrations of TG and total cholesterol (TC) were measured using enzymatic tests. At the same time, as the physical examination, the estimated glomerular filtration rate (eGFR), high- and low-density lipoprotein-cholesterol (HDL- and LDL-C) levels, and blood pressure were all measured. The averages were applied if the participant's blood pressure was measured more than once.

### Mortality outcomes

By looking up the ICD-10 numbers I00-I078 for each cause of death among study participants, a clear classification of all causes of death was achieved. Cardiovascular events, including mortality from heart illnesses and cerebrovascular disorders, were this study's main outcome variables. All-cause mortality was the secondary research outcome in this investigation. As of 31 December 2019, participants in the NHANES from 1999–2018 had mortality follow-up data.

### Statistical analyses

The NHANES-recommended weights were successfully applied to the study analysis. ANOVA was used to statistically assess continuous variables, which are reported as the mean standard error (SE). Using the chi-square approach, categorical variables are assessed as percentages.

To establish the optimal TyG index, restricted cubic spline regression analysis was used. Log-rank tests and standard Kaplan‒Meier plots were used to evaluate survival. Using Cox proportional hazards models based on proposed weights, hazard ratios (HRs) and 95% confidence intervals (CIs) for cardiovascular events and all-cause mortality were estimated. Model 1 had a basic design. Age, gender, and race were the only factors in Model 2 that were accounted for. Other confounders, such as age, sex, race, education level, alcohol use, cigarette smoking status, BMI, LDL-C level, HDL-C level, eGFR, family history of DM, family history of CVD, hypertension, DM, and CVD, were also taken into account when adjusting Model 3. The predictive significance of the TyG index for cardiovascular events and all-cause mortality in obese and nonobese people was also assessed in this investigation.

A two-sided *P* value of 0.05 indicated statistical significance. Data analysis was carried out using the R program (version 4.2.0; Vienna, Austria).

## Results

The basic traits of the two groups were categorized after dividing individuals based on the TyG index (Table 1). The TyG_H group's participants tended to be older, male, and overweight (all *P* < 0.001). In addition, SBP, DBP, FPG, LDL-C, and TG levels, the percentage of smokers, and the prevalence of pre-DM, DM, hypertension, and CVD were all higher in the high TyG index group (all *P* < 0.001).

**Table 1:** Bas eline according to the level of TyG

**Characteristics**	**TyG_L** (TyG < 8.5)	**TyG_H** (TyG ≥ 8.5)	***P*-value**
	*N* = 7979	*N* = 8634	
**Age(year)**	38.3 ± 0.2	43.6 ± 0.2	< 0.001
**Female, n (%)**	4616 (57.8)	3784 (42.6)	< 0.001
**Race, n (%)**			< 0.001
Mexican American	1221 (7.6)	2061 (10.8)	
Non-Hispanic Black	2280 (16.0)	1246 (7.8)	
Non-Hispanic White	2995 (63.6)	3620 (67.8)	
Other Hispanic	637 (5.6)	853 (6.2)	
Other Race	846 (7.2)	854 (7.5)	
**Education level, n (%)**			< 0.001
< 12	1439 (13.4)	2385 (18.7)	
12	2542 (37.9)	2864 (39.9)	
≥ 12	3428 (48.7)	3179 (41.4)	
**Smoke status, n (%)**	2846 (39.8)	4160 (50.6)	< 0.001
**Alcohol using, n (%)**	5915 (89.2)	6915 (90.8)	0.008
**BMI (kg/m**^**2**^**)**	26.8 ± 0.1	30.6 ± 0.1	< 0.001
**SBP (mmHg)**	115.6 ± 0.2	121.8 ± 0.2	< 0.001
**DBP (mmHg)**	69.2 ± 0.2	73.4 ± 0.2	< 0.001
**FPG (mg/dl)**	94.7 ± 0.2	110.4 ± 0.5	< 0.001
**LDL-C (mg/dl)**	107.5 ± 0.5	124.6 ± 0.5	< 0.001
**HDL-C (mg/dl)**	59.4 ± 0.3	46.8 ± 0.2	< 0.001
**TG (mg/dl)**	69.1 ± 0.3	186.3 ± 2.0	< 0.001
**eGFR (ml/min)**	103.2 ± 0.4	98.2 ± 0.3	< 0.001
**Family CVD, n (%)**	803 (11.1)	1196 (15.1)	< 0.001
**Family DM, n (%)**	3000 (37.3)	4083 (46.1)	< 0.001
**Hypertension, n (%)**	2622 (30.5)	4649 (53.4)	< 0.001
**CVD, n (%)**	268 (2.9)	650 (6.5)	< 0.001
**DM, n (%)**	335 (3.2)	1868 (17.0)	< 0.001
**Pre-DM, n (%)**	634 (7.5)	1551 (17.6)	< 0.001

*TyG* Triglyceride-glucose, *BMI* Body mass index, *SBP* Systolic blood pressure, *DBP* Diastolic blood pressure, *FPG* Fasting plasma glucose, HDL-C High-density lipoprotein cholesterol, *LDL-C* Low-density lipoprotein cholesterol, *TG* Triglyceride, *eGFR* Estimated glomerular filtration rate, *CVD* Cardiovascular Disease, DM Diabetes mellitus

The TyG index and body habitus were used to further classify the participants (obese vs. nonobese). The patients were older and had the highest percentages of pre-DM, DM, hypertension, and CVD in the TyG_H and obese groups (all *P* < 0.001, Table 2). The TyG_H and obese groups also had the greatest rates of DM and CVD in their families. After stratification by dyslipidemia and diabetes, a higher TyG score was significantly associated with higher cardiovascular events and all-cause mortality in both nondiabetic and non dyslipidemic populations (eTable 1).

**Table 2:** Baseline according to TyG and different obesity states

Characteristics	TyG_L/non-obesity	TyG_L/obesity	TyG_H/non-obesity	TyG_H/obesity	*P*-value
	*N* = 5896	*N* = 2083	*N* = 4663	*N* = 3971	
**Age**	37.9 ± 0.3	39.7 ± 0.4	42.9 ± 0.3	44.4 ± 0.3	< 0.001
**Female, n (%)**	3223(55.6)	1393(65.0)	1788(38.6)	1996(47.3)	< 0.001
**Race, n (%)**					< 0.001
Mexican American	904(7.1)	317(9.2)	1108(10.4)	953(11.2)	
Non-Hispanic Black	1372(12.5)	908(27.4)	531(6.1)	715(9.8)	
Non-Hispanic White	2404(66.7)	591(53.4)	1927(67.4)	1693(68.2)	
Other Hispanic	475(5.4)	162(6.0)	467(6.7)	386(5.7)	
Other Race	741(8.2)	105(4.0)	630(9.4)	224(5.2)	
**Education level, n (%)**					< 0.001
< 12	1047(13.0)	392(14.7)	1301(18.8)	1084(18.7)	
12	1893(39.2)	649(33.6)	1584(42.0)	1280(37.5)	
≥ 12	2465(47.8)	963(51.7)	1638(39.3)	1541(43.8)	
**Smoke status, n (%)**	2142(41.0)	704(36.1)	2300(52.6)	1860(48.4)	< 0.001
**Alcohol using, n (%)**	4376(90.0)	1539(86.7)	3750(91.7)	3165(89.8)	< 0.001
**BMI (kg/m**^**2**^**)**	24.0 ± 0.1	35.9 ± 0.2	25.8 ± 0.1	36.2 ± 0.1	< 0.001
**SBP (mmHg)**	114.0 ± 0.3	120.9 ± 0.4	119.5 ± 0.3	124.5 ± 0.3	< 0.001
**DBP (mmHg)**	68.5 ± 0.2	71.7 ± 0.4	72.3 ± 0.2	74.9 ± 0.3	< 0.001
**FBG (mg/dl)**	93.8 ± 0.2	97.9 ± 0.4	105.3 ± 0.6	116.3 ± 0.7	< 0.001
**LDL (mg/dl)**	106.1 ± 0.6	112.2 ± 0.8	126.1 ± 0.7	122.9 ± 0.8	< 0.001
**HDL (mg/dl)**	61.0 ± 0.3	54.1 ± 0.4	49.0 ± 0.3	44.1 ± 0.2	< 0.001
**TG (mg/dl)**	68.3 ± 0.4	71.8 ± 0.6	180.4 ± 2.5	193.2 ± 3.0	< 0.001
**eGFR (ml/min)**	103.0 ± 0.4	104.2 ± 0.6	98.6 ± 0.4	97.7 ± 0.4	< 0.001
**Family CVD, n (%)**	543(10.5)	260(12.9)	550(12.9)	646(17.7)	< 0.001
**Family DM, n (%)**	2026(34.7)	974(45.6)	1915(39.9)	2168(53.3)	< 0.001
**Hypertension, n (%)**	1581(25.5)	1041(46.7)	2078(43.7)	2571(64.8)	< 0.001
**CVD, n (%)**	165(2.6)	103(3.7)	258(4.8)	392(8.5)	< 0.001
**DM, n (%)**	150(1.9)	185(7.3)	702(10.5)	1166(24.7)	< 0.001
**Pre-DM, n (%)**	386(6.3)	248(11.2)	715(14.2)	836(21.6)	< 0.001

*TyG* Triglyceride-glucose, *BMI* Body mass index, *SBP* Systolic blood pressure, *DBP* Diastolic blood pressure, *FPG* Fasting plasma glucose, *HDL-C* High-density lipoprotein cholesterol, *LDL-C* Low-density lipoprotein-C, *TG* Triglyceride, *eGFR* Estimated glomerular filtration rate, *CVD*, Cardiovascular Disease, *DM* Diabetes mellitus

**Fig. 2:**
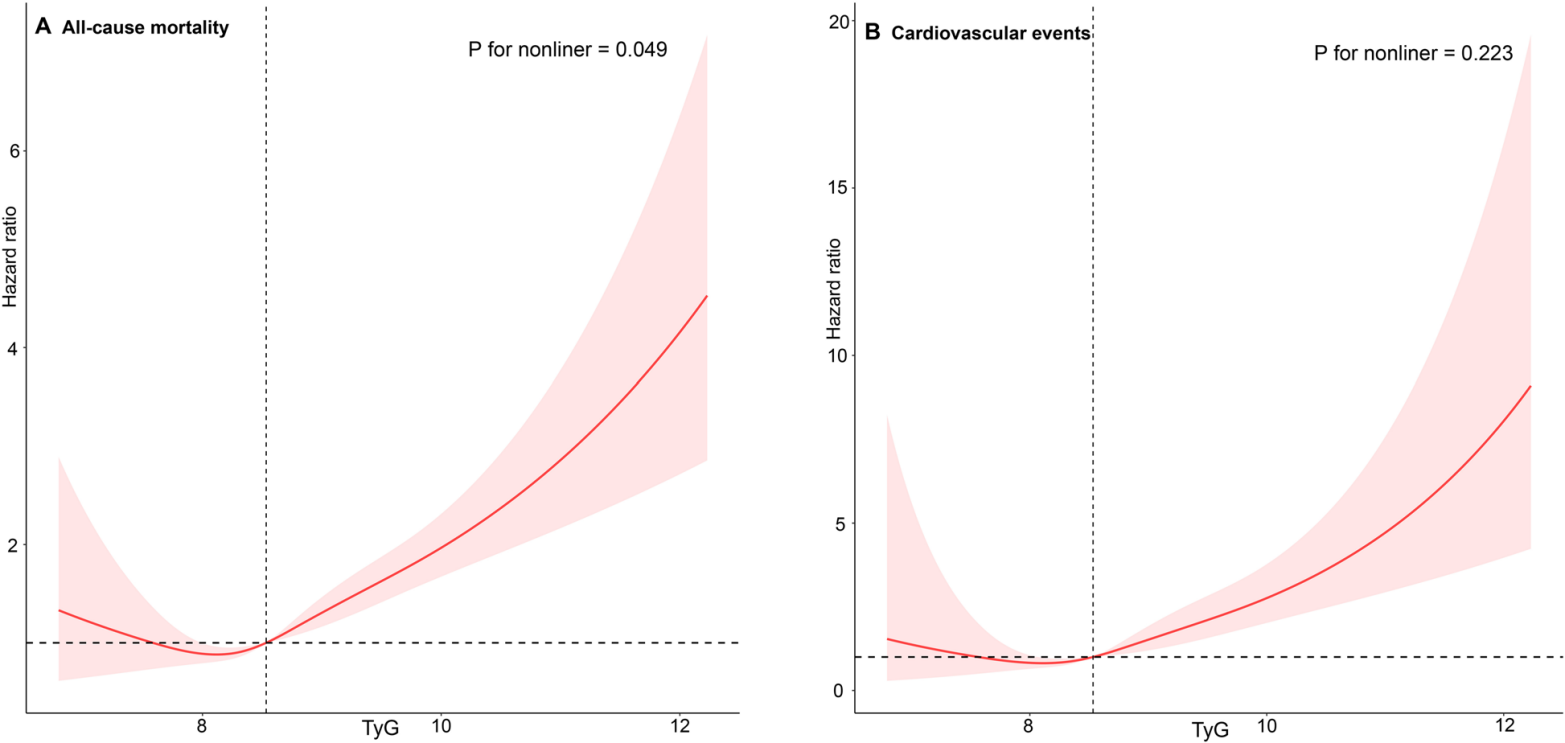
Hazard ratios for all-cause mortality and cardiovascular events based on restricted cubic spine function for the TyG index

**Fig. 3:**
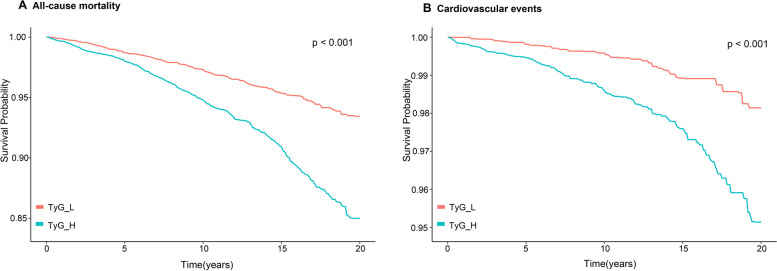
Kaplan‒Meier analysis for all-cause mortality and cardiovascular events according to different TyG indices

A 123-month median follow-up period was used. The best TyG index cutoff value was 8.5 for both all-cause mortality and cardiovascular events, according to restricted cubic spline analysis (Fig. 2) (all-cause mortality: *P* value nonlinear = 0.049; cardiovascular events: *P value nonlinear* = 0.223). The participants' incidence of cardiovascular events or all-cause death was strongly correlated with the TyG index (all *P* < 0.01; Fig. 3). A high TyG index increased the risk of cardiovascular events by 63% (*P* = 0.040; Table 3) and the risk of all-cause mortality by 32% (*P* = 0.010) in individuals after adjusting for all covariates. The results from the analysis of death from all causes were equivalent to those from cardiovascular events.

**Table 3:** The HR (95% CI) of All-cause mortality and Cardiovascular event according to TyG from the three models

Characteristics	Model 1		Model 2		Model 3	
	**HR (95% CI)**	***P*-value**	**HR (95% CI)**	***P*-value**	**HR (95% CI)**	***P*-value**
**All-cause mortality**						
TyG_L	Reference	-	Reference	-	Reference	-
TyG_H	2.11 (1.76,2.52)	< 0.001	1.48(1.23,1.78)	< 0.001	1.32 (1.07,1.63)	0.010
**Cardiovascular event**						
TyG_L	Reference	-	Reference	-	Reference	-
TyG_H	2.80(1.92,4.08)	< 0.001	1.89 (1.28,2.80)	0.001	1.63 (1.03,2.58)	0.040

Model 1: Not adjusted. Model 2: Adjusted for age, sex and race. Model 3: Adjusted for age, sex, race, education level, alcohol using, smoking status, body mass index, low-density lipoprotein cholesterol high-density lipoprotein cholesterol, eGFR, family diabetes mellitus, family cardiovascular disease, hypertension, diabetes mellitus, cardiovascular diseases

*CI* Confidence interval, *TyG* Triglyceride-glucose index, *HR* Hazard ratio

**Fig. 4:**
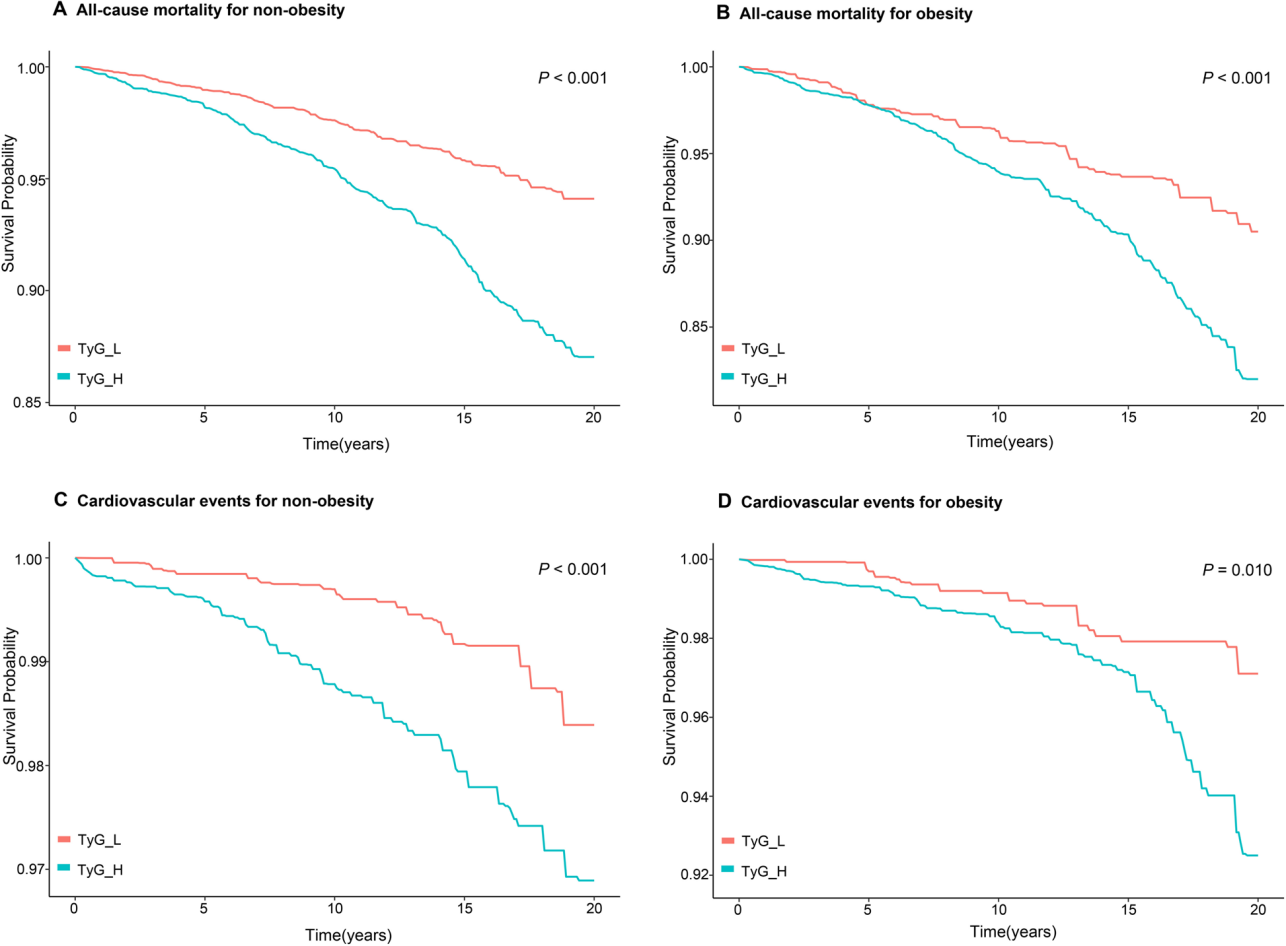
Kaplan‒Meier analysis for all-cause mortality according to different TyG indices for nonobese participants (**A**) and obese participants (**B**) and cardiovascular events according to different TyG indices for nonobese participants (**C**) and obese participants (**D**)

**Fig. 5:**
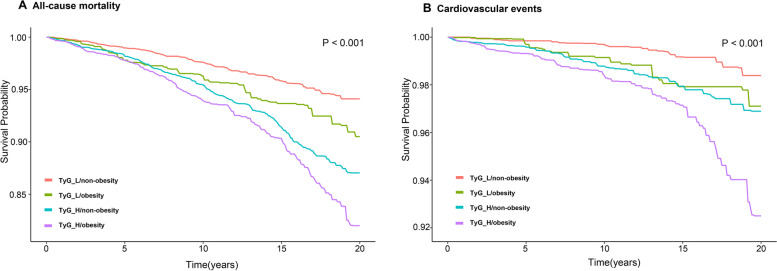
Kaplan‒Meier curve analysis for all-cause mortality and cardiovascular events according to the TyG index and body habitus (obese *vs*. nonobese)

The Kaplan‒Meier curves revealed that the TyG groups had dramatically different rates of cardiovascular events or all-cause mortality as a result of obesity (all *P* < 0.05; Fig. 4). After adjusting for all covariates, a high TyG index was increased by 1.41 flod to cardiovascular events among obese people (*P* = 0.020; Table 4). The nonobese individuals in Model 3 did not significantly differ between the TyG groups (*P* = 0.08). After excluding patients with DM, the results were more significant than in the previous analysis (TyG_H/obesity: HR, 2.96 CI, 1.26,6.95; *P* = 0.010; eTable 2). In comparison to the other groups, the high TyG and obese group had the highest probability of a cardiovascular event and dying from any cause; furthermore, compared with the TyG_L/obesity group, the TyG_H/nonobesity highest group had significantly higher all-cause mortality (all *P* < 0.001; Fig. 5).

**Table 4:** The HR (95% CI) of All-cause mortality and Cardiovascular event according to TyG and different obesity states from the three models

Characteristics	Model 1		Model 2		Model 3	
	**HR (95% CI)**	***P*-value**	**HR (95% CI)**	***P*-value**	**HR (95% CI)**	***P*-value**
**All-cause mortality**						
TyG_L/non-obesity	Reference	-	Reference	-	Reference	-
TyG_L/obesity	1.61 (1.17,2.23)	0.004	1.40 (1.01,1.96)	0.004	1.41 (0.94,2.14)	0.100
TyG_H/non-obesity	2.14 (1.73,2.64)	< 0.001	1.48 (1.19,1.83)	< 0.001	1.35 (1.07,1.71)	0.010
TyG_H/obesity	2.68 (2.14,3.34)	< 0.001	1.77 (1.41,2.21)	< 0.001	1.51 (1.07,2.12)	0.020
**Cardiovascular event**						
TyG_L/non-obesity	Reference	-	Reference	-	Reference	-
TyG_L/obesity	2.17(1.12,4.20)	0.020	1.86 (0.95,3.61)	0.070	2.00 (0.84,4.74)	0.120
TyG_H/non-obesity	2.59 (1.56,4.31)	< 0.001	1.68 (1.02,2.79)	0.040	1.66 (0.94,2.95)	0.080
TyG_H/obesity	4.60 (2.86,7.38)	< 0.001	2.92 (1.81, 4.72)	< 0.001	2.41 (1.13,5.12)	0.020

Model 1: Not adjusted. Model 2: Adjusted for age, sex and race. Model 3: Adjusted for age, sex, race, education level, alcohol using, smoking status, body mass index, low-density lipoprotein cholesterol, high-density lipoprotein cholesterol, eGFR, family diabetes mellitus, family cardiovascular disease, hypertension, diabetes mellitus, cardiovascular diseases

*CI* Confidence interval, *TyG* Triglyceride-glucose index, *HR* Hazard ratio

## Discussion

In this retrospective analysis, it was found that cardiovascular event risk and long-term all-cause mortality in young and middle-aged adults were positively correlated with a high TyG index. Moreover, those who were obese and had a high TyG index were more likely to experience cardiovascular problems. Nevertheless, in nonobese individuals, this study confirmed that a high TyG index was associated with an increased risk for cardiovascular events.

Reduced sensitivity and reactivity to the effects of insulin characterize the IR state. Individuals with IR are more prone to metabolic conditions, such dysglycemia, dyslipidemia, and hypertension, which all have a negative impact on patients with CVD prognoses [17]. When used to assess IR in both diabetic and nondiabetic individuals, the TyG index was identified as more reliable than homeostasis model assessment of IR [7]. TyG was later recognized to play a crucial role in the progression of CVD in diabetic and nondiabetic patients and in the prognosis of CVD patients [18]. According to consistent clinical data, patients with CAD have a worse prognosis when their TyG index is increased [19, 20]. According to the current study, the TyG index may be useful for risk classification and prognosis prediction in acute coronary syndrome patients with or without diabetes [21, 22]. Liu et al. [23] discovered that the TyG level was nonlinearly correlated with cardiovascular mortality in individuals, including those without CVD or DM. There is no information regarding how to utilize the TyG index in younger participants, and the bulk of studies using the TyG index in patients with CVD have been done on middle-aged and older people [12]. This investigation filled this gap and showed that cardiovascular event risk and long-term all-cause mortality were positively correlated with a high TyG index in young and middle-aged adults. However, this result is based only on the NHANES database analysis. NHANES is a widely recognized and reputable source of health data in the United States that provides comprehensive and reliable information on a wide range of health indicators. However, the use of NHANES data allows for comparison with other studies conducted in international comparisons. However, it is not always appropriate to extrapolate findings from the U.S. data to other countries because the two countries have different demographic and environmental factors that may affect health outcomes. Therefore, more studies in different countries are needed in the future to validate the reliability of the results.

The TyG index impact on cardiovascular events does not have a known underlying mechanism. The TyG index, a reflection of IR, consists of two CVD risk factors (associated components with lipid and glucose levels). According to previous studies, IR causes an increase in free radicals and glycosylation products, which inactivates nitric oxide (NO). Endothelium-dependent vasodilation is brought on by the aberrant NO secretion associated with IR, which also affects the vascular endothelium [24]. Moreover, IR causes excessive generation of reactive oxygen species, which also damages endothelial cells [25]. These effects are brought on by IR activation of the mitochondrial electron transport chain. Blood free fatty acid (FFA) levels have a significant role in the emergence of inflammation and IR linked to obesity [26]. The majority of obese populations have elevated plasma FFA levels, and while IR is decreased by an increase in plasma FFA levels, the expression of proinflammatory cytokines is enhanced as well. Moreover, lipoprotein (a) (Lp(a)) has been recognized as an important risk factor in patients with premature CAD [27, 28]. Lp(a) was also found to have an inverse association with IR by inducing atherogenicity through proinflammatory activities [29, 30]. The TyG index exhibits a stronger association with Lp(a) levels than HOMA-IR [31]. This may also be one of the potential mechanisms of the TyG index on cardiovascular events.

There is another point worth mentioning. TyG is composed of triglycerides and glucose. When we further analyzed the association of TyG with all-cause mortality and cardiovascular events stratified by dyslipidemia and diabetes, we found that higher TyG was significantly associated with higher cardiovascular events and worse all-cause mortality in both nondiabetic and nondyslipidemic populations. The predictive value of TyG for IR was in the nondiabetic population but not in the diabetic population, and we speculated that anti-diabetic treatment was one of the reasons for the poor predictive performance of TyG. In addition, Hameed Ekhlas Khalid. et al. [32] found that TyG was significantly elevated in diabetic patients with poor glycemic control, which may partially impair the ability of TyG to predict the prognosis of diabetic populations with poor glycemic control. Likewise, elevated triglyceride levels lead to an increase in free fatty acids, which increases the flux of free fatty acids from adipose to nonadipose tissue, which may affect glycemic control [33]; higher levels of triglycerides in liver and muscle may affect various glucose metabolism in target organs [34, 35]. Therefore, we have reason to believe that TyG is reliable in predicting cardiovascular events and all-cause mortality in nondiabetic and nondyslipidemic populations.

### Comparisons with other studies and what does the current work add to the existing knowledge

Excess body fat is referred to as obesity, which is a significant risk factor for DM and CVD. Currently, BMI is a reliable indicator for diagnosing obesity clinically; however, a growing body of evidence argues that BMI alone is insufficient to diagnose obesity, as it does not provide a body fat distribution that is more relevant to IR and clinical outcomes [36, 37]. The TyG index has been added to BMI as a replacement indicator of the distribution of body fat. Combining BMI and IR is crucial for predicting future cardiovascular events in community populations. Recent research, however, has shown contradictory findings about the link between obesity and the TyG index. According to Hou et al. [38], the connection between BMI and stroke prognosis was unaffected by the TyG index, and obese individuals with ischemic stroke fared better than low- or normal-weight patients in terms of survival. According to Fritz et al. [39], more than half of the correlation between BMI and end-stage renal disease in the general population can be attributed to the TyG index and the likelihood of developing the condition. It is also critical to understand that TyG mediates the effect of greater BMI on the risk of gastrointestinal malignancies. Another investigation discovered links between the TyG index and the likelihood of developing these cancers [40]. In keeping with earlier studies, the current study found that obesity and cardiovascular events were highly connected to a high TyG index in young and middle-aged people. Nevertheless, we failed to find a meaningful connection in the group of people who were not obese.

It may be advantageous to include IR assessment and therapies in long-term management plans for young and middle-aged community-dwelling adults, as previous research has clarified the impact of IR on future cardiovascular events in these populations. In fact, studies on IR therapies in this population are limited, so it is unclear whether IR therapies are necessary for treating these patients. However, we noticed that diverse dietary patterns have a significant protective effect on IR and inflammatory markers [15, 16, 41] Interestingly, a mediation analysis showed that adiposity partially mediated the connection between whole-grain consumption and IR, which may be the basis for this conclusion [15]. Another study reported that reducing abdominal obesity may play an important role in the pathway by which consumption of the Mediterranean diet reduces IR and inflammation [16]. These studies have emphasized that dietary structure and IR or indicators of inflammation are influenced by obesity. Our study found that obesity status strengthens the relationship between IR and poor cardiovascular outcome. Therefore, public health efforts, including but not limited to dietary pattern adjustment, to reduce body weight may reduce the cardiovascular burden of IR. Moreover, the effect of different dietary patterns on the TyG index deserves further study.

## Study strengths and limitations

### Strengths

This study uses the TyG index as an indication of IR to assess CVD events in young and middle-aged individuals with and without obesity. It also provides clinical evidence that obesity and IR jointly impair cardiovascular prognosis in this population. Second, the assessment of fasting lipids and glucose is strictly regulated by NHANES. Finally, to reduce mistakes, these data were corrected for actual or possible confounders.

## Limitations

This study had some limitations. First, the TyG index was not actively evaluated in individuals during the follow-up timespan owing to the retrospective design. Second, because we did not conduct a mediating effects study, it is important to interpret the data with care. Third, due to missing data on statin and hypoglycemic agent use during follow-up, the effect of intervention with lipid-lowering therapy and hypoglycemic agents on mortality outcome during follow-up could not be excluded. Finally, the sampling of NHANES data left nearly 50% of fasting glucose values missing. However, by analyzing the NHANES recommended weights, the study results are equally accurate in reflecting the true status of this population.

## Conclusion

This study found that cardiovascular events in young and middle-aged community-dwelling persons were independently correlated with the TyG index. Moreover, the strength of this connection grew with obese status. To reduce future cardiovascular events in this population, it is critical that IR be considered in the evaluation and treatment of young and middle-aged obese individuals.

## Supplementary Information

**Additional file 1:** **eTable 1** The HR (95% CI) of All-cause mortality and Cardiovascular event of different TyG groups stratified by diabetes and dyslipidemia.

**Additional file 2:** **eTable 2** The HR (95% CI) of All-cause mortality and Cardiovascular event according to TyG and different obesity states from the three models, exclusion of DM participant.

## Data Availability

Not applicable.

## Abbreviations

TyG: Triglyceride-glucose
TyG_H: High-level triglyceride-glucose
TyG _L: Low-level triglyceride-glucose
CVD: Cardiovascular disease
IR: Insulin resistance
DM: Diabetes mellitus
TG: Fasting triglycerides
NHANES: The National Health and Nutrition Examination Survey
NCHS: The National Center for Health Statistics
CDC: The Centers for Disease Control and Prevention
BMI: Body mass index
FPG: Fasting plasma glucose
TC: Total cholesterol
HDL-C: High-density lipoprotein-cholesterol
LDL-C: Low-density lipoprotein-cholesterol
SBP: Systolic blood pressure
DBP: Diastolic blood pressure
eGFR: Estimated glomerular filtration rate
ICD-10: The International Classification of Diseases 10^th^ edition
SE: Standard error
ANOVA: Analysis of variance
HRs: Hazard ratios
CIs: Confidence intervals
NO: Nitric oxide
FFA: Free fatty acid
