# Supplementary material for: Association between the insulin resistance marker TyG index and subsequent adverse long-term cardiovascular events in young and middle-aged US adults based on obesity status
Source: Lipids Health Dis. 2023 May 18;22:65. doi: 10.1186/s12944-023-01834-y (PMC10193684; doi:10.1186/s12944-023-01834-y)
Supplement: Supplementary file 1 — Additional file 1: eTable 1 The HR (95% CI) of All-cause mortality and Cardiovascular event of different TyG groups stratified by diabetes and dyslipidemia. [file 12944_2023_1834_MOESM1_ESM.docx]

**eTable 1. The HR (95% CI) of All-cause mortality and Cardiovascular event of different TyG groups stratified by diabetes and dyslipidemia.**

|  | **All-cause mortality** | |  | **Cardiovascular event** | |
| --- | --- | --- | --- | --- | --- |
|  | **HR (95% CI)** | **P-value** |  | **HR (95% CI)** | **P-value** |
| **Diabetes** |  | |  |  | |
| TyG_L | Reference | - |  | Reference | - |
| TyG_H | 1.50 (0.83,2.70) | 0.180 |  | 1.01 (0.33,3.09) | 0.980 |
| **Non-Diabetes** |  | |  |  | |
| TyG_L | Reference | - |  | Reference | - |
| TyG_H | 1.25(1.00,1.58) | 0.050 |  | 1.70(1.00,1.92) | 0.050 |
|  | | | | | |
| **Dyslipidemia** |  |  |  |  |  |
| TyG_L | Reference | - |  | Reference | - |
| TyG_H | 1.20(0.92,1.57) | 0.170 |  | 1.35(0.78,2.32) | 0.280 |
| **Non-Dyslipidemia** |  |  |  |  |  |
| TyG_L | Reference | - |  | Reference | - |
| TyG_H | 1.58(1.03,2.40) | 0.030 |  | 2.75(1.16,6.51) | 0.020 |

Adjusted for age, sex, race, education level, alcohol using, smoking status, body mass index, low-density lipoprotein cholesterol high-density lipoprotein cholesterol, eGFR, family diabetes mellitus, family cardiovascular disease, hypertension, cardiovascular diseases.

CI, Confidence interval; TyG, Triglyceride-glucose index; HR, Hazard ratio.
