# Supplementary material for: Association between the insulin resistance marker TyG index and subsequent adverse long-term cardiovascular events in young and middle-aged US adults based on obesity status
Source: Lipids Health Dis. 2023 May 18;22:65. doi: 10.1186/s12944-023-01834-y (PMC10193684; doi:10.1186/s12944-023-01834-y)
Supplement: Supplementary file 2 — Additional file 2: eTable 2 The HR (95% CI) of All-cause mortality and Cardiovascular event according to TyG and different obesity states from the three models, exclusion of DM participant. [file 12944_2023_1834_MOESM2_ESM.docx]

**eTable 2. The HR (95% CI) of All-cause mortality and Cardiovascular event according to TyG and different obesity states from the three models, exclusion of DM participant.**

| **Characteristics** | **Model 1** | |  | **Model 2** | |  | **Model 3** | |
| --- | --- | --- | --- | --- | --- | --- | --- | --- |
|  | **HR (95% CI)** | **P-value** |  | **HR (95% CI)** | **P-value** |  | **HR (95% CI)** | **P-value** |
| **All-cause mortality** |  | |  |  | |  |  | |
| TyG_L/non-obesity | Reference | - |  | Reference | - |  | Reference | - |
| TyG_L/obesity | 1.43 (1.03,1.98) | 0.003 |  | 1.29 (0.93,1.80) | 0.130 |  | 1.68 (1.05,2.69) | 0.030 |
| TyG_H/non-obesity | 1.90 (1.51,2.38) | <0.001 |  | 1.38 (1.09,1.73) | 0.010 |  | 1.37 (1.07,1.76) | 0.010 |
| TyG_H/obesity | 1.95 (1.51,2.51) | <0.001 |  | 1.38 (1.07,1.78) | 0.010 |  | 1.63 (1.07,2.49) | 0.020 |
| **Cardiovascular event** |  | | | | | | | |
| TyG_L/non-obesity | Reference | - |  | Reference | - |  | Reference | - |
| TyG_L/obesity | 2.00 (0.98,4.08) | 0.060 |  | 1.81 (0.86,3.82) | 0.120 |  | 2.42 (0.93,6.27) | 0.070 |
| TyG_H/non-obesity | 2.32 (1.30,4.16) | 0.005 |  | 1.58 (0.88,2.81) | 0.120 |  | 1.95 (1.03,3.69) | 0.040 |
| TyG_H/obesity | 3.11 (1.85,5.22) | <0.001 |  | 2.11 (1.27,3.51) | 0.004 |  | 2.96 (1.26,6.95) | **0.010** |

Model 1: Not adjusted. Model 2: Adjusted for age, sex and race. Model 3: Adjusted for age, sex, race, education level, alcohol using, smoking status, body mass index, low-density lipoprotein cholesterol, high-density lipoprotein cholesterol, eGFR, family diabetes mellitus, family cardiovascular disease, hypertension, cardiovascular diseases.

CI, Confidence interval; TyG, Triglyceride-glucose index; HR, Hazard ratio.
